# Ceruloplasmin Deficiency Reduces Levels of Iron and BDNF in the Cortex and Striatum of Young Mice and Increases Their Vulnerability to Stroke

**DOI:** 10.1371/journal.pone.0025077

**Published:** 2011-09-16

**Authors:** Sarah J. Texel, Jian Zhang, Simonetta Camandola, Erica L. Unger, Dennis D. Taub, Raymond C. Koehler, Z. Leah Harris, Mark P. Mattson

**Affiliations:** 1 Department of Neuroscience, Johns Hopkins University School of Medicine, Baltimore, Maryland, United States of America; 2 Laboratory of Neurosciences, National Institute on Aging Intramural Research Program, Baltimore, Maryland, United States of America; 3 Department of Anesthesiology and Critical Care Medicine, Johns Hopkins University School of Medicine, Baltimore, Maryland, United States of America; 4 Department of Nutrition Sciences, Pennsylvania State University, University Park, Pennsylvania, United States of America; 5 Laboratories of Immunology, National Institute on Aging, National Institutes of Health, Baltimore, Maryland, United States of America; 6 Department of Pediatrics, Vanderbilt University, Nashville, Tennessee, United States of America; Hokkaido University, Japan

## Abstract

Ceruloplasmin (Cp) is an essential ferroxidase that plays important roles in cellular iron trafficking. Previous findings suggest that the proper regulation and subcellular localization of iron are very important in brain cell function and viability. Brain iron dyshomeostasis is observed during normal aging, as well as in several neurodegenerative disorders such as Alzheimer's, Parkinson's and Huntington's diseases, coincident with areas more susceptible to insults. Because of their high metabolic demand and electrical excitability, neurons are particularly vulnerable to ischemic injury and death. We therefore set out to look for abnormalities in the brain of young adult mice that lack Cp. We found that iron levels in the striatum and cerebral cortex of these young animals are significantly lower than wild-type (WT) controls. Also mRNA levels of the neurotrophin brain derived neurotrophic factor (BDNF), known for its role in maintenance of cell viability, were decreased in these brain areas. Chelator-mediated depletion of iron in cultured neural cells resulted in reduced BDNF expression by a posttranscriptional mechanism, suggesting a causal link between low brain iron levels and reduced BDNF expression. When the mice were subjected to middle cerebral artery occlusion, a model of focal ischemic stroke, we found increased brain damage in Cp-deficient mice compared to WT controls. Our data indicate that lack of Cp increases neuronal susceptibility to ischemic injury by a mechanism that may involve reduced levels of iron and BDNF.

## Introduction

Iron is an essential nutrient that plays an important role in myelination, energy production and cell cycling; also it serves as a cofactor for enzymes involved in neurotransmitter production and metabolism, DNA synthesis and purine catabolism. [1], [2]. All of these actions are mediated by the transition metal property of iron that allows it to act as an electron donor or acceptor. Unfortunately, the same property that makes iron so useful can also lead to cell damage through formation of hydroxyl radicals when ferrous iron interacts with hydrogen peroxide in the Haber-Weiss-Fenton reaction [3]. Regulation of iron homeostasis is thus extremely important because of its dual nature [4]. One of the proteins involved in controlling iron trafficking is the essential ferroxidase ceruloplasmin (Cp), which acts to convert the reactive ferrous (Fe^2+^) form of iron to the much less toxic ferric (Fe^3+^) form , therefore facilitating transferrin-mediated transport [5].

Cp is an abundant plasma protein also found in the brain in both a secreted form and a glycosylphosphatidylinositol (GPI)-linked isoform on the plasma membrane of astrocytes [6]. Patients affected by aceruloplasminemia lack Cp and develop progressive iron accumulation in organs such as the liver, pancreas and retina; as these patients age, iron also accumulates in the brain, particularly in the basal ganglia and substantia nigra, which results in neuronal degeneration and neurological symptoms [7]. Similarly Cp knockout (CpKO) mice develop age-dependent iron deposits in the cerebellum, brain stem and cervical spinal cord leading to neurological deficits [8]. Experiments have demonstrated that the absence of Cp in both humans [9], [10] and mice [11], [12] render neurons more susceptible to insults, suggesting a role for Cp in neuronal survival.

Increases in free iron levels have been observed in the brain during global [13] and focal ischemia [14]. The role played by iron in ischemic brain injury is complex. The ability of iron chelators or antioxidants treatments to reduce ischemic brain injury has generally been linked to the capacity of Fe^2+^ to induce lipid peroxidation and oxidative damage [15]–[17]. However, additional mechanisms are likely involved. Iron is critical for the function of numerous enzymes involved in neuronal metabolism, plasticity and survival. For example, mitochondria rely upon heme containing cytochromes (complexes III–IV) and on iron-sulfur clusters containing complexes (I–III), in the electron transport chain and enzymes of the citric acid cycle [18]. Because mitochondrial function is compromised in stroke, reduced iron levels may exacerbate ischemia-related cellular energy deficits. Also, enzymes involved in the production and metabolism of monoamine neurotransmitters also require iron [19], and two of these neurotransmitters (serotonin and norepinephrine) have been reported to modify brain damage in animal models of stroke [20], [21]

In response to cerebral ischemia the production of several neurotrophic factors, including fibroblast growth factor 2, insulin-like growth factor 1 and brain-derived neurotrophic factor (BDNF) is increased [22]–[24]. Activation of receptors for each of the latter trophic factors can protect neurons against ischemic brain injury in experimental cell culture and animal models [25]–[28]. The expression of BDNF by neurons is sensitive to iron levels as demonstrated by studies showing that dietary iron deficiency reduce BDNF expression without affecting expression of the BDNF receptor TrkB [29]. Also in patients with neurodegenerative diseases, such as Parkinson's, Huntington's and Alzheimer's, brain iron dysregulation is found together with decreases in BDNF [30]. BDNF administration has been has also been shown to promote cell viability after insults [31] and the increased BDNF production after exercise and caloric restriction is believed to play a major role in the neuroprotective effects of these treatments [32].

In the present study we demonstrate that iron levels are reduced in the cerebral cortex and striatum of young adult CpKO mice, and that the reduction in iron is associated with reduced BDNF expression. CpKO mice exhibit increased brain damage in a model of focal ischemic stroke, suggesting an important role for cellular iron homeostasis in neuronal resistance to ischemic injury.

## Results

### Ceruloplasmin deficiency results in reduced concentrations of iron in the cerebral cortex and striatum

**Figure 1:**
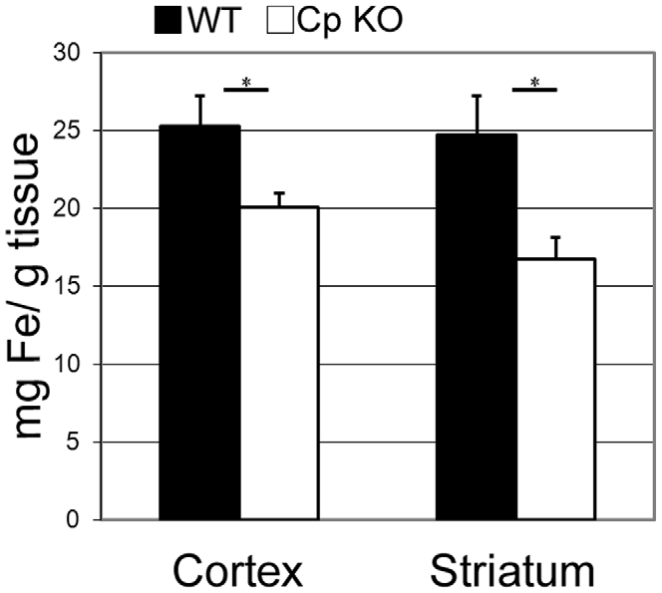
CpKO mice have decreased concentrations of iron in the cerebral cortex and striatum. Atomic absorption measures of cortical and striatal biopsy punches show significant decreases in iron concentrations in CpKO compared to wild type mice in both the cerebral cortex ( n = 18; *p<0.02) and the striatum (n = 7; *p<0.03).

Cp plays a key role in iron regulation. To determine if the absence of Cp altered brain iron levels, biopsy punches were taken from the cerebral cortex and striatum of young 3 month-old WT and CpKO mice. These punches were analyzed for iron levels using atomic absorption spectroscopy. Levels of iron were normalized to the sample weight. The CpKO mice had a significantly lower concentration of iron in both the cortex and striatum compared to WT mice (Fig. 1). This result is very interesting considering that 3 month-old CpKO mice already display a hepatic iron overload phenotype [33]. The discrepancy between brain and peripheral iron levels is likely due to the tight control exerted by the brain blood barrier in regulating cerebral iron influx/efflux [34].

### BDNF expression is reduced in the cerebral cortex and striatum of ceruloplasmin-deficient mice

**Figure 2:**
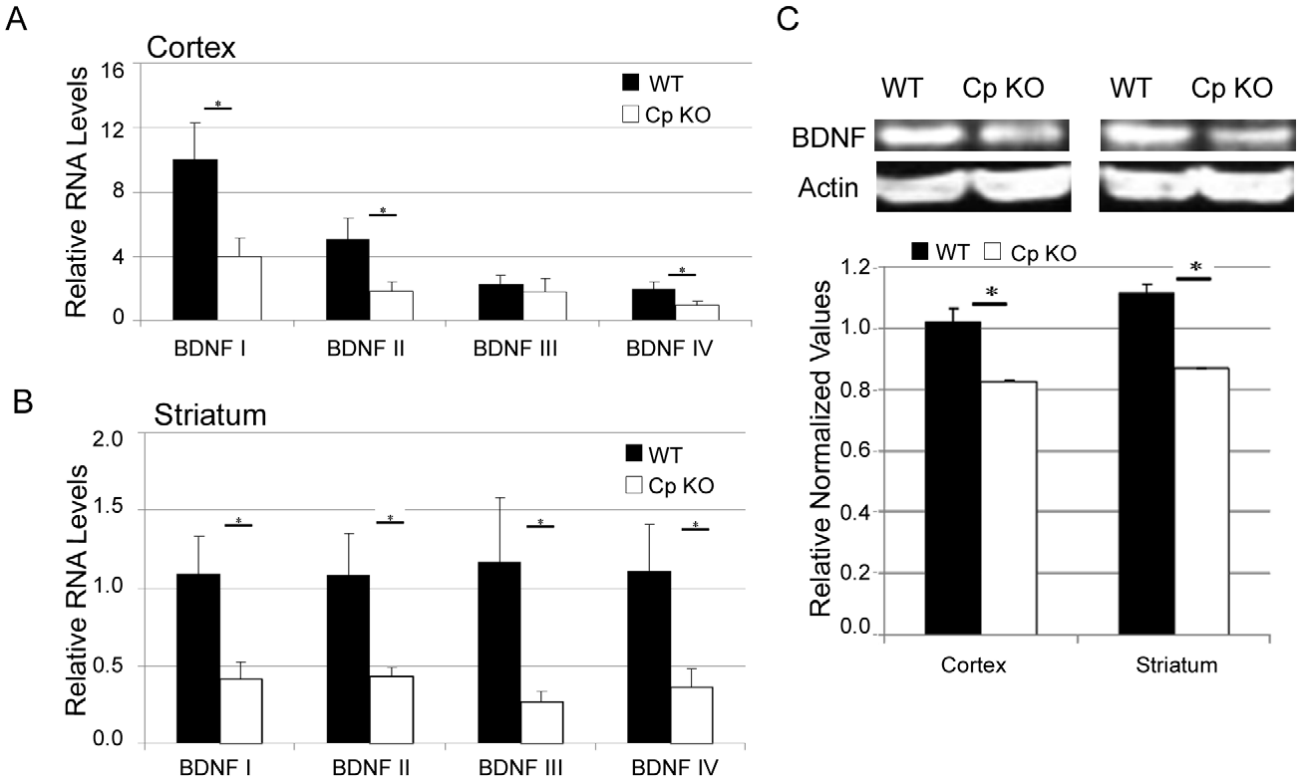
The expression of BDNF is reduced in the cerebral cortex and striatum of ceruloplasmin-deficient mice. **A.** RT-PCR measures of the 4 BDNF transcripts show a decrease in BDNF transcripts 1, 2 and 4 in the cortex of the CpKO mouse (*p<0.04, n = 5). **B.** RT-PCR measures of the 4 BDNF transcripts show a decrease in BDNF transcripts 1, 2, 3 and 4 in the striatum of the CpKO mouse (*p<0.05, n = 5). **C.** Examples of immunoblots of BDNF protein levels in the cortex and striatum (top panel) and densitometry results showing significant decreases BDNF protein levels (normalized to actin level) using Image J software. (*p<0.03; n = 4 WT and 3 CpKO mice).

There are several different BDNF transcripts produced in the mouse. Their expression is stimulated through various mechanisms, but all result in the same final BDNF protein [35]. Using real-time PCR we measured levels of 4 different transcripts of BDNF in the cerebral cortex and striatum of young 3 month old CpKO mice. We found that levels of BDNF transcripts I, II and IV were significantly reduced in the cortex of CpKO mice compared to 3 month old WT mice (Fig. 2A). Levels of BDNF transcripts I, II, III and IV were significantly reduced in the striatum of the CpKO mice compared to WT controls (Fig. 2B). Measurements of the transcripts of both short and long forms of the BDNF receptor TrkB did not show differences between WT and Cp KO animals in either the cortex or striatum (data not shown). Western blots also demonstrated a significant decrease in BDNF protein levels in both the striatum and cortex in the CpKO compared to WT animals (Fig. 2C).

**Figure 3:**
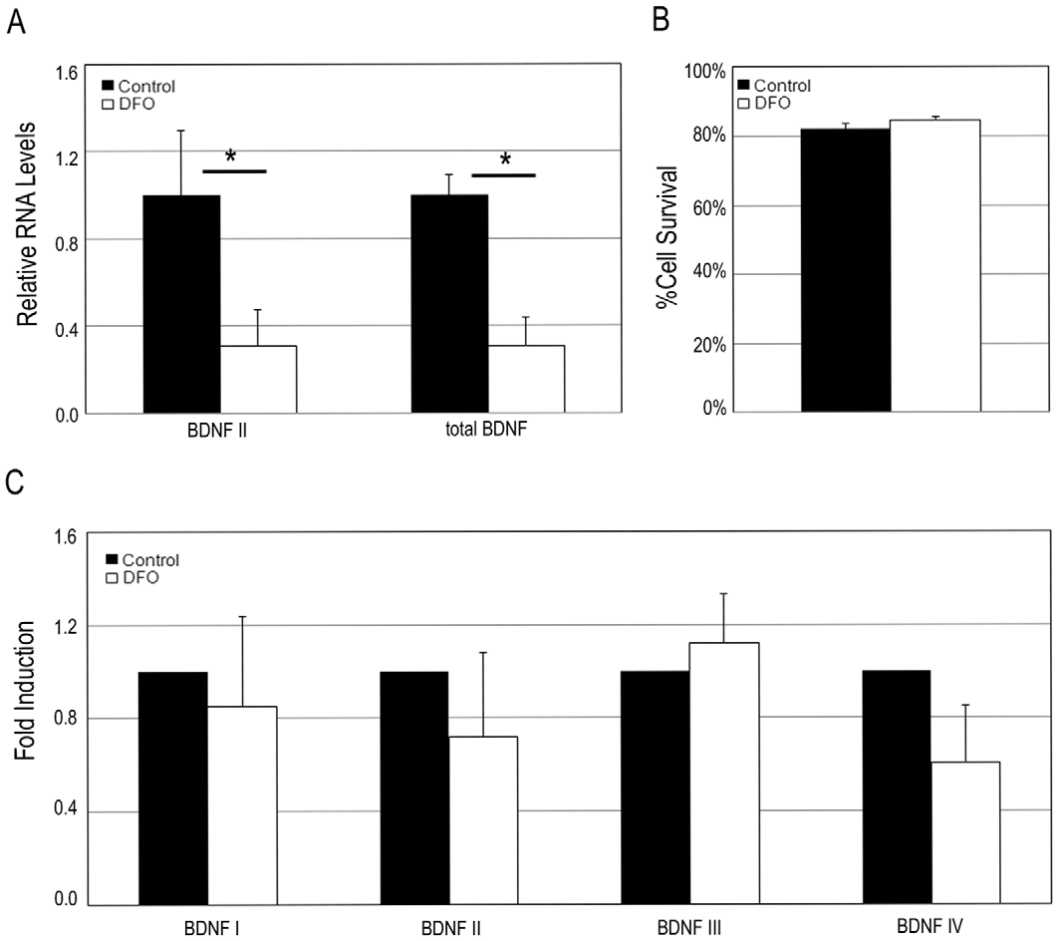
Iron chelation decreases BDNF expression in human neuroblastoma cells. **A.** SH-SY5Y cells were treated with either vehicle or 100 µM deferoxamine (DFO) for 24 hours. RT-PCR measures of BDNF transcripts show a decrease in total BDNF transcripts (tBDNF, variants 1–14, 16–18 (*p<0.01, n = 4–5). **B.** Iron chelation did not cause changes in cell survival as assessed by trypan blue exclusion 24 hours post-treatment (n = 5). **C.** SH-SY5Y cells were transfected with luciferase reporters for the promoter regions of BDNF I–IV and were then treated with 100 µM deferoxamine for 24 hours. No significant differences were observed between control and treated cells (n = 3–4).

To better explore the connection between iron and BDNF levels we employed a cell culture model (human SH-SY5Y neuroblastoma cells). The cells were treated for 24 hours with the iron chelator deferoxamine. Similar to what was observed in vivo in CpKO mice, decreasing iron availability resulted in a significant decrease of BDNF transcript levels (Fig. 3A) without effecting cell viability (Fig. 3B). When SH-SY5Y cells were transfected with reporters for BDNF promoters I–IV and then subjected to iron chelation, no differences in reporter activity were observed amongst control or deferoxamine treated cells (Fig. 3C). Taken together, our data suggest that iron regulates BDNF at a post-transcriptional level, possibly by accelerating the decay of BDNF mRNAs.

### CpKO mice sustain greater brain damage after MCAO

**Figure 4:**
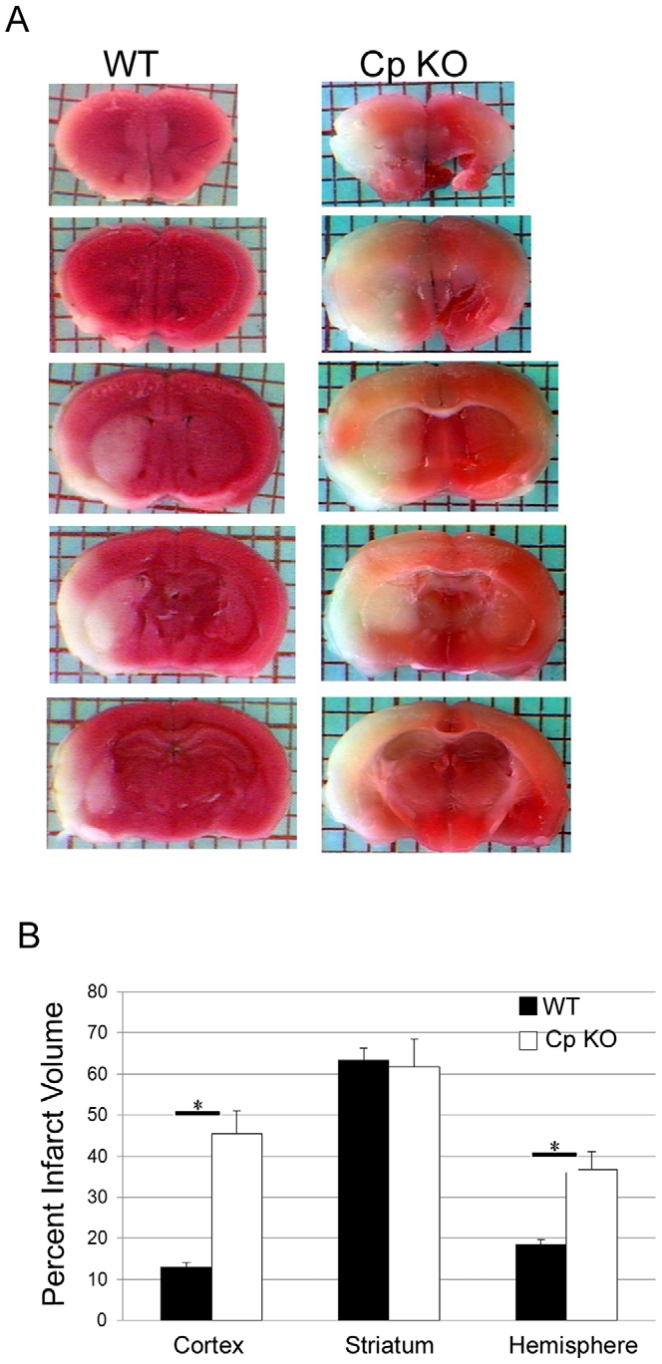
CpKO mice have increased cerebral infarct volume after focal ischemic stroke. **A.** Representative images of TTC-stained brain sections in mice subjected to 45 min MCAO with a 72 hours post-stroke survival. **B.** Calculation of percent infarct volume show a significant increase in the cortex and hemisphere of the CpKO mice compared to WT mice. (*p<0.002; n = 8 WT and 6 KO mice).

Three month old WT and CpKO mice were subjected to 45 minutes of MCAO followed by 72 hours of reperfusion. Infarct volume was measured by image analysis of TTC-stained brain sections (Fig. 4A). We found that cell death in the striatum was extensive and there was no significant difference in the amount of damage between CpKO and WT mice. However, the extent of cell death in the cerebral cortex was significantly greater in the CpKO mice compared to WT mice (Fig. 4B). These results may be explained by the fact that the striatum has less collateral blood flow than cortex [36]; consequently 45 minutes of ischemia produces near maximal damage in the striatum but submaximal damage in the cortex. Thus, in our experimental model the “core” striatal damage is maximal and not impacted by CP deficiency. On the other hand, in the cortical “penumbra” region the reduced levels of total iron and BDNF in the CpKO mouse render the neurons more susceptible to death after the ischemic insult exacerbating the stroke outcome.

**Figure 5:**
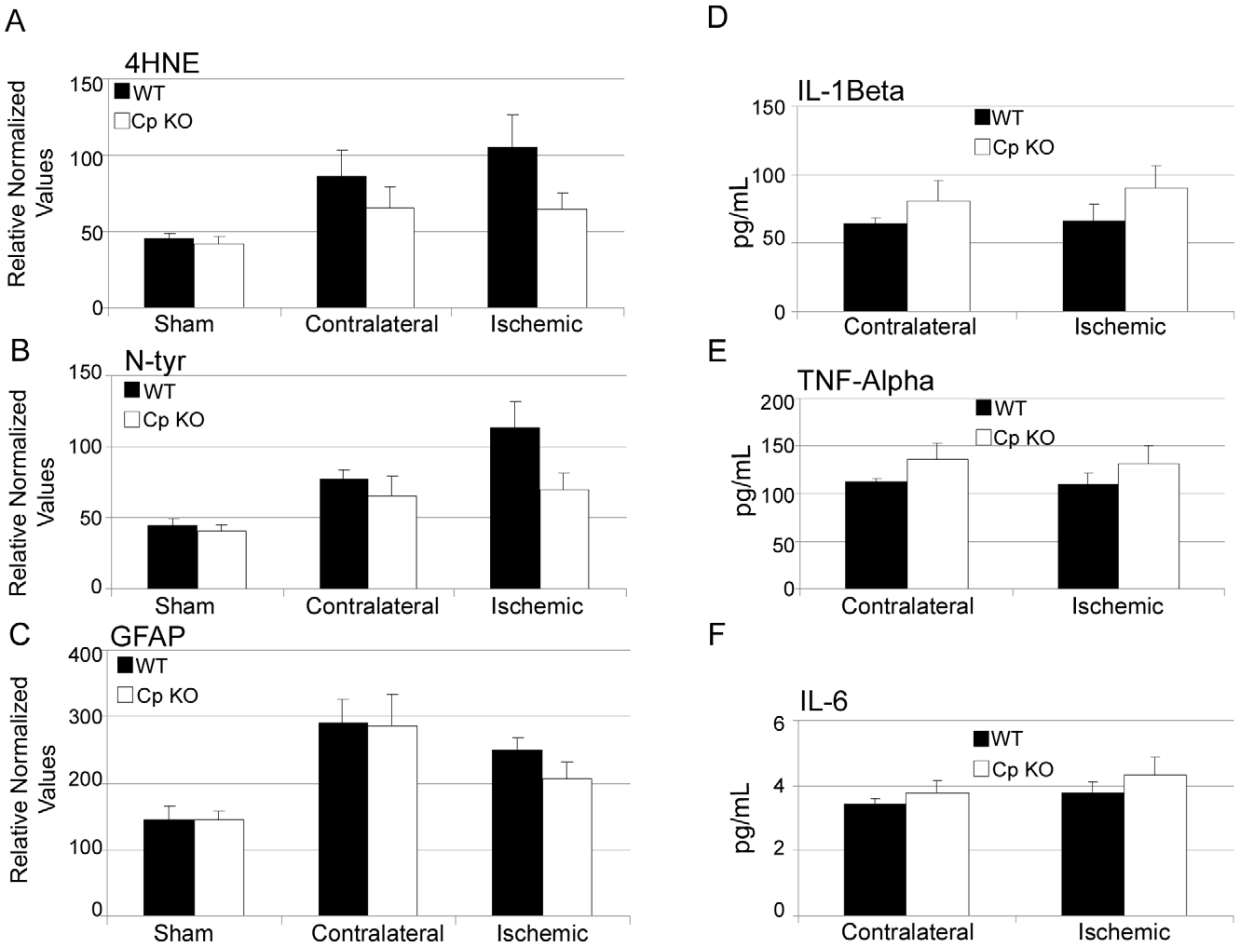
CpKO mice do not exhibit increased levels of oxidative stress, glial reactivity or cytokine levels in the cerebral cortex. **A–C.** Densitometry measures of 4-hydroxynonenal (4HNE) (A), Nitrotyrosine (B) and GFAP (C) in the cortex of sham operated animals and MCAO operated animals from the ischemic or contralateral side (24 hours after reperfusion). No differences were seen between genotypes (n = 4). Protein levels were normalized to actin. **D–F.** Bio-Plex measurements of the cytokines IL-1β, TNF-α and IL-6 in pg/mL in the cortex of CpKO and WT animals 3 hours after reperfusion (n = 7 per group). No significant differences were seen between the two groups.

Because Fe^2+^ may promote the generation of reactive oxygen species (ROS), which can lead to lipid peroxidation and peroxynitrite production [37], we measured markers of oxidative stress that have previously been associated with ischemic brain injury. After 24 hours reperfusion, the lipid peroxidation product 4-hydroxynonenal and nitrotyrosines were elevated in both the ipsilateral and contralateral cerebral hemisphere in WT mice subjected to stroke compared to sham-operated control mice (Fig. 5A, B). Similar results were obtained in CpKO mice with no significant differences in levels of 4-hydroxynonenal or nitrotyrosine between WT and CpKO mice, although trends towards reduced levels of these markers were observed in the ipsilateral cortex of CpKO compared to WT mice (Fig. 5A, B). In addition, we found that levels of GFAP, a marker of reactive astrocytes, were increased in both hemispheres in response to stroke with no significant differences between WT and CpKO mice (Fig. 5C). Levels of proinflammatory cytokines (IL-1Beta, TNF-alpha and IL-6) measured in brain tissue samples collected 3 hours after reperfusion were also not significantly different between WT and CpKO mice in either hemisphere (Fig. 5D, E, F). These data further support the hypothesis that the decreases in iron and BDNF in the CpKO mouse contributes to the larger infarct volume, rather than differences in levels of oxidative stress and inflammation.

## Discussion

Ceruloplasmin is a key regulator in iron metabolism and its loss has been shown to cause age-dependent iron dysregulation in humans and animal models [6], [33], [38]. We found that total iron levels in the cerebral cortex and striatum of young CpKO mice were significantly lower than WT mice. This iron deficit was concurrent with decreased levels of BDNF mRNA and protein. The extent of brain damage caused by MCAO reperfusion was significantly greater in the CpKO mice compared to WT mice, as a result of the expansion of the damage in the ischemic penumbra region of the cerebral cortex. These findings suggest that lack of Cp can cause a change in brain iron and BDNF levels, which normally play important roles in protecting neurons against ischemic brain injury.

Previous studies of other lines of CpKO mice have shown that iron levels increase in the brain stem, cerebellum and spinal cord as they age [8], [38]. However, levels of iron in striatum and cerebral cortex, from young CpKO mice have not been reported in the latter studies. The larger infarct volume we observed in the CpKO mice after MCAO, suggests that lack of Cp may affect the vulnerability of cortical and striatal neurons. While the present study is the first to examine the effects of ceruloplasmin deficiency on the vulnerability of the brain to ischemic stroke, Rathore et al. [12] demonstrated that young CpKO mice show increased secondary damage coupled with oxidative damage and a decrease in functional recovery after spinal cord injury compared to WT animals. Previous evidence has also shown enhanced levels of oxidative stress markers in the brain of patients with aceruloplasminemia [39], [40], as well as in the hippocampus of CpKO mouse compared to WT following 4 weeks of rotenone treatment [11]. However, in our experimental model we found no evidence of increased lipid peroxidation or astrocyte reactivity the in cortex or striatum of young CpKO mice compared to WT mice measured 24 hours after the insult. We also did not observe increased proinflammatory cytokine levels 3 hours after the insult. These findings suggest that Cp deficiency and reduced iron levels do not alter levels of membrane-associated oxidative stress or generation of proinflammatory cytokines by glial cells. However, we cannot exclude the possibility that Cp and/or iron levels modify oxidative stress and inflammatory processes during the later post-stroke period.

The majority of the literature on cerebral ischemia and iron indicate that increased levels of iron contributing to the exacerbation of infarct volume or functional recovery. Elevated iron levels may contribute to ischemic brain injury by promoting the production of hydroxyl radicals and consequent lipid peroxidation [41]. Human studies have also shown that increased iron stores are associated with a worse outcome after stroke [42]. Studies involving iron chelators have demonstrated reduced infarct size with both pre and post [43]–[45]. The mechanisms in which chelators promote protection though, may not be due just to their reduction of free iron but to other factors such as HIF activation [46], [47] or reduction of brain edema [41]. Toxicity from iron during ischemia likely arises from liberation of iron from high-molecular weight storage proteins [13]. We found decreased concentrations of iron in the cortex and striatum of CpKO mice in conjunction with increased infarct volumes. This counterintuitive result may be due to the particular importance of iron in the brain. While high levels of iron may be toxic, it is also true that abnormally low levels of iron will make cells more sensitive to insults by impairing normal brain functions which rely on adequate iron supply, such as ATP production, regulation of cellular energy, myelination and neurotransmitter production [41]. Interestingly, others have shown that increases in infarct volume after MCAO in rats on high iron diets were not associated with alterations in brain iron levels [48]. Consistently, aggressive iron chelator treatment in rats was not able to reduce infarct volume following ischemia [49]. This supports our hypothesis that reduced brain iron may increase neuronal vulnerability.

Previous studies have demonstrated that BDNF can protect neurons against cerebral ischemic damage in animal models [50]–[52] and against more specific insults relevant to ischemic stroke including glucose deprivation, excitotoxicity and oxidative stressors [53]–[55]. BDNF is known to promote the plasticity and survival of neurons, playing key roles in adaptive responses of the brain to environmental challenges [56]. Studies in rats have shown that cerebral ischemia can differently affect BDNF levels in the core, where a decrease occurs, and penumbra areas where an increase occurs [57], supporting a role for protection by BDNF. Indeed studies in both mice and rats have demonstrated that administration of exogenous BDNF can promote a reduction in infarct volume and functional recovery after cerebral ischemia [58], [59], [52]. Similarly, studies using interventions known to up regulate basal BDNF levels, such as dietary restriction, enriched environment or exercise, have shown decreased infarct volumes following MCAO [60]–[63]. Conversely decreasing BDNF levels or attenuating its effects following cerebral ischemia diminishes recovery of function [64], [65]. BDNF has been shown to exert anti-apoptotic and neuroplastic properties, as well as to enhance neuro- and angio-genesis. There is also evidence that BDNF levels can modulate cortical excitability with lower levels of BDNF enhancing excitability [66]. More specifically, lack of BDNF transcript IV has been show to cause impairment in cortical inhibitory signaling [67]. Our finding of reduced BDNF transcript levels, including transcript IV, in the CpKO mice suggests a mechanism whereby perturbed cellular iron metabolism in brain cells results in reduced BDNF levels in neurons which renders them vulnerable to ischemic injury. Indeed, we found that chelation of cellular iron in results in a reduction in BDNF mRNA levels in cultured neural cells. The lack of changes in BDNF promoter-driven luciferase activity following deferoxamine, suggests that the reduction in BDNF mRNA is likely the result of decreased mRNA stability. Notably, iron has been shown to play a role in the regulation of transcript stability in various experimental models by modifying the ability of iron-responsive proteins to bind to specific stabilizing/destabilizing sequences at the 3′ and/or 5′ of mRNA untranslated regions [68], [69].

Because aceruloplasminemia's most striking symptoms manifest late in life, studies looking at iron brain levels in pre-symptomatic young patients, or in young rodent models of aceruloplasminemia may provide novel insight into the roles of cellular iron metabolism in developmental neuroplasticity and disease vulnerability. Our data clearly show that the lack of Cp leads to an early-on reduction in total brain iron and BDNF levels, resulting in increased vulnerability of neurons to ischemic injury .

## Materials and Methods

### Mice

Ceruloplasmin knock-out (CpKO) mice were generated as described previously [33]. Wild type (WT) and CpKO strains were maintained according to National Institutes of Health and Johns Hopkins University guidelines. All procedures on animals were approved by the Animal Care and Use Committee of the National Institute on Aging Intramural Research Program. In addition, procedures for producing middle cerebral artery occlusion (MCAO) were approved by the Animal Care and Use Committee of Johns Hopkins University.

### Brain iron analysis

Brain regions (n = 16/group) were wet digested in 0.2% ultra-pure nitric acid using standard procedures and analyzed for iron concentration by atomic absorption spectrometry (Perkin Elmer Analyst 600, Perkin Elmer, Norwalk, CT) [70]. Standards were prepared by diluting a Perkin Elmer iron standard (PE#N9300126) in 0.2% ultra-pure nitric acid, and blanks were prepared with digesting and diluting reagents to control for possible contamination. All standard curves exceeded r>0.99.

### RNA extraction and real-time PCR

RNA was isolated using Trizol (Invitrogen) and purified with an RNA Micro Kit (Qiagen, Valencia, CA). Following treatment with DNAse I, RNA was quantified and equal amounts were retro-transcribed using the SuperScript First Strand Synthesis System (Invitrogen Life Technologies). Real-time PCR analysis was performed with a PTC 200 Pelthier Thermo Cycler and Chromo 4 Fluorescent Detector (BioRad, Hercules, CA), and Sybr® Green PCR Master Mix according to the manufacturer's instructions (Applied Biosystems, Foster City, CA). Each reaction included 3 µl of diluted (1∶4) cDNA and was performed in triplicate. PCR was performed under the following conditions: 10 min at 95°C, followed by 40 cycles of 30 s at 95°C, 30 s at 60°C and 1 min at 72°C. The comparative Ct method was used to determine the normalized changes of the target gene relative to a calibrator reference. The primers used in this study were as follows: mBDNF1 (NM_007540), 5′- GCT TTG CGG ATA TTG CGA AGG GTT -3′ and 5′- ACC TGG TGG AAC ATT GTG GCT TTG -3′; mBDNF2 (NM_001048139), 5′- TGA AGT TGG CTT CCT AGC GGT GTA -3′ and 5′- TGG TGG AAC TTC TTT GCG GCT TAC -3′; mBDNF3 (NM_001048141) 5′- CCA GAG CAG CTG CCT TGA TGT TTA -3′ and 5′- CCG CCT TCA TGC AAC CGA AGT AT -3′; mBDNF4 (NM_001048142), 5′- TGA CAA CAA TGT GAC TCC ACT GCC -3′ and 5′- ATG GTC ATC ACT CTT CTC ACC TGG -3′; tBDNF (X91251.1 ) 5′- AGA AGA GCT GTT GGA TGA GGA CCA-3′ and 5′- AGG CTC CAA AGG CAC TTG ACT ACT-3′; HPRT (NM_013556), 5′- CCT GCT GGA TTA CAT TAA AGC ACT G-3′ and 5′- CCT GAA GTA CTC ATT ATA GTC AAG G-3′.

### Cell transfection and reporter assay

Human neuroblastoma SH-SY5Y cells were transfected with 1 µg of BDNF(I–IV)-driven luciferase reporter and 0.2 µg of pRL-TK vector (Promega, Madison, WI) expressing renilla luciferase using Fugene 6 (Roche, Nutley, NJ). Luciferase activity was quantified using a Dual Luciferase Reporter System 24 hours post transfection.

### Immunoblot Analysis

Proteins were extracted from cortex or striatum in WT (n = 4) or CpKO (n = 3) mouse brain tissue; 60 µg of protein was separated by Novex Bis-Tris PAGE (4–12%) and then transferred to a PVDF membrane. The membrane was blocked in 5% non-fat milk for 1 hour at room temperature, followed by an overnight incubation at 4°C with primary antibodies against: actin (Sigma); BDNF (Santa Cruz); 4-hydroxynonenal [71] nitrotyrosine (Millipore); GFAP (Sigma). Membranes were then washed and incubated with secondary antibodies for 1 hour at room temperature. Protein bands were visualized using a chemiluminescence detection kit (Amersham Biosciences, Piscataway, NJ, USA). Densitometry measurements were made using Image J software and protein levels were normalized to actin.

### Mouse model of MCAO

Mice were housed in a room at a temperature of 22±1°C and 12 hours dark and light cycle. Standard chow pellets and water was allowed ad libitum. CpKO and wild type mice (22–28 g) were anesthetized with isoflurane (induction with 2∼5% and maintained on 1%). The concentration of inspired oxygen was adjusted to 25–30%. Body temperature was maintained at 37±0.5°C using a heating pad during the surgery. Mice underwent 45 min MCAO with 72 hours of reperfusion by the standard trans-luminal method, which has been described in previous reports [72]. Briefly, the right common carotid artery (CCA) was exposed through a 15 mm medial line incision of the neck beneath the jaw. The external carotid artery (ECA) was carefully separated from the adjacent vagus nerve and tissues and isolated for a length of 3–5 mm away from the bifurcation of CCA, and the distal end was coagulated and cut off. The proximal end of CCA was temporarily ligated using a 6-0 suture. Then a 7-0 nylon monofilament coated with silicone was inserted from the distal end of the isolated ECA and was gently introduced into the internal carotid artery and advanced approximately 6–8 mm past the carotid bifurcation to the origin of the middle cerebral artery (MCA). Successful MCAO was confirmed by decreased cerebral blood flow in the territory of the MCA measured by laser-Doppler flowmetry (LDF; Moor Instruments Ltd.; Model MBF3D). The onset of ischemia was designated at the time when the blood flow had decreased to below 30% of the baseline blood flow. The suture remained in situ for 45 min, and then reperfusion of the MCA was initiated by withdrawal of the filament and release of the CCA ligature. Mice were euthanized 72 hours after the stroke and infarct volumes were quantified using standard volumetric analysis with correction for swelling. The brain was chilled and the cerebrum was sliced into 5 pieces of 1 mm coronal sections. The slices were then incubated in 1% triphenyltetrazolium choloride (TTC) in phosphate buffer, and stained at 37°C for 10–15 min. Both anterior and posterior views of 5 coronal sections were captured with a digital camera and the infarct areas were traced using the SigmaScan Pro, Image analysis, Version 5.0 (SPSS Inc.). Infarct volume was analyzed on 8 WT and 6 CpKO brains.

### Levels of Cytokines

Murine cytokine levels from CpKO (n = 7) and WT (n = 7) mice were analyzed using Bio-Plex Mouse Cytokine Singleplex kits according to the manufacturer's instructions (Biorad Laboratories, Hercules, CA). The results are expressed as pg/mL and all assays were run in duplicate.

### Statistical Analysis

Statistical significance was determined using the Student's t-test with a p-value less than 0.05 proving statistical significance. Error bars represent the standard error.

## References

[1] Valerio LG (2007). Mammalian iron metabolism.. Toxicol Mech Methods.

[2] Zhang AS, Enns CA (2009). Iron homeostasis: recently identified proteins provide insight into novel control mechanisms.. J Biol Chem.

[3] Mattson MP (2004). Metal-catalyzed disruption of membrane protein and lipid signaling in the pathogenesis of neurodegenerative disorders.. Ann N Y Acad Sci.

[4] Chua AC, Graham RM, Trinder D, Olynyk JK (2007). The regulation of cellular iron metabolism.. Crit Rev Clin Lab Sci.

[5] Hellman NE, Gitlin JD (2002). Ceruloplasmin metabolism and function.. Annu Rev Nutr.

[6] Harris ZL, Klomp LW, Gitlin JD (1998). Aceruloplasminemia: an inherited neurodegenerative disease with impairment of iron homeostasis.. Am J Clin Nutr.

[7] Xu X, Pin S, Gathinji M, Fuchs R, Harris ZL (2004). Aceruloplasminemia: An inherited neurodegenerative disease with impairment of iron homeostasis.. Ann N Y Acad Sci.

[8] Patel BN, Dunn RJ, Jeong SY, Zhu Q, Julien JP (2002). Ceruloplasmin regulates iron levels in the CNS and prevents free radical injury.. J Neurosci.

[9] Texel SJ, Xu X, Harris ZL (2008). Ceruloplasmin in neurodegenerative diseases.. Biochem Soc Trans.

[10] Vassiliev V, Harris ZL, Zatta P (2005). Ceruloplasmin in neurodegenerative diseases.. Brain Res Brain Res Rev.

[11] Kaneko K, Hineno A, Yoshida K, Ikeda S (2008). Increased vulnerability to rotenone-induced neurotoxicity in ceruloplasmin-deficient mice.. Neurosci Lett.

[12] Rathore KI, Kerr BJ, Redensek A, Lopez-Vales R, Jeong SY (2008). Ceruloplasmin protects injured spinal cord from iron-mediated oxidative damage.. J Neurosci.

[13] Lipscomb DC, Gorman LG, Traystman RJ, Hurn PD (1998). Low molecular weight iron in cerebral ischemia acidosis in vivo.. Stroke.

[14] Millerot-Serrurot E, Bertrand N, Mossiat C, Faure P, Prigent-Tessier A (2008). Temporal changes in free iron levels after brain ischemia. Relevance to the timing of iron chelation therapy in stroke.. Neurochem Int.

[15] Hall ED (1992). Novel inhibitors of iron-dependent lipid peroxidation for neurodegenerative disorders.. Ann Neurol.

[16] Palmer C, Roberts RL, Bero C (1994). Deferoxamine posttreatment reduces ischemic brain injury in neonatal rats.. Stroke.

[17] Hanson LR, Roeytenberg A, Martinez PM, Coppes VG, Sweet DC (2009). Intranasal deferoxamine provides increased brain exposure and significant protection in rat ischemic stroke.. J Pharmacol Exp Ther.

[18] Gille G, Reichmann H (2010). Iron-dependent functions of mitochondria-relation to neurodegeneration.. J Neural Transm.

[19] Youdim MB, Green AR (1978). Iron deficiency and neurotransmitter synthesis and function.. Proc Nutr Soc.

[20] Bielenberg GW, Burkhardt M (1990). 5-hydroxytryptamine1A agonists. A new therapeutic principle for stroke treatment.. Stroke.

[21] Windle V, Power A, Corbett D (2007). Norepinephrine depletion facilitates recovery of function after focal ischemia in the rat.. Eur J Neurosci.

[22] Gluckman P, Klempt N, Guan J, Mallard C, Sirimanne E (1992). A role for IGF-1 in the rescue of CNS neurons following hypoxic-ischemic injury.. Biochem Biophys Res Commun.

[23] Kumon Y, Sakaki S, Kadota O, Matsuda S, Fujita H (1993). Transient increase in endogenous basic fibroblast growth factor in neurons of ischemic rat brains.. Brain Res.

[24] Kokaia Z, Zhao Q, Kokaia M, Elmér E, Metsis M (1995). Regulation of brain-derived neurotrophic factor gene expression after transient middle cerebral artery occlusion with and without brain damage.. Exp Neurol.

[25] Cheng B, Mattson MP (1991). NGF and bFGF protect rat hippocampal and human cortical neurons against hypoglycemic damage by stabilizing calcium homeostasis.. Neuron.

[26] Mattson MP, Cheng B (1993). Growth factors protect neurons against excitotoxic/ischemic damage by stabilizing calcium homeostasis.. Stroke.

[27] Jiang N, Finklestein SP, Do T, Caday CG, Charette M (1996). Delayed intravenous administration of basic fibroblast growth factor (bFGF) reduces infarct volume in a model of focal cerebral ischemia/reperfusion in the rat.. J Neurol Sci.

[28] Lee HJ, Lim IJ, Lee MC, Kim SU (2010). Human neural stem cells genetically modified to overexpress brain-derived neurotrophic factor promote functional recovery and neuroprotection in a mouse stroke model.. J Neurosci Res.

[29] Tran PV, Carlson ES, Fretham SJ, Georgieff MK (2008). Early-life iron deficiency anemia alters neurotrophic factor expression and hippocampal neuron differentiation in male rats.. J Nutr.

[30] Zuccato C, Cattaneo E (2009). Brain-derived neurotrophic factor in neurodegenerative diseases.. Nat Rev Neurol.

[31] Marini AM, Jiang X, Wu X, Pan H, Guo Z (2007). Preconditioning and neurotrophins: A model for brain adaptation to seizures, ischemia and other stressful stimuli.. Amino Acids.

[32] Mattson MP, Duan W, Wan R, Guo Z (2004). Prophylactic activation of neuroprotective stress response pathways by dietary and behavioral manipulations.. NeuroRx.

[33] Harris ZL, Klomp LW, Gitin JD (1999). Targeted gene disruption reveals an essential role for ceruloplasmin in cellular iron efflux.. Proc Natl Acad Sci USA.

[34] Rouault TL, Cooperman S (2006). Brain iron metabolism.. Semin Pediatr Neurol.

[35] Aid T, Kazantseva A, Piirsoo M, Palm K, Timmusk T (2007). Mouse and rat BDNF gene structure and expression revisited.. J Neurosci Res.

[36] Kiewert C, Mdzinarishvili A, Hartmann J, Bickel U, Klein J (2010). Metabolic and transmitter changes in core and penumbra after middle cerebral artery occlusion in mice.. Brain Res.

[37] Hall ED, Detloff MR, Johnson K, Kupina NC (2004). Peroxynitrite-mediated protein nitration and lipid peroxidation in a mouse model of traumatic brain injury.. J Neurotrauma (United States).

[38] Yamamoto K, Yoshida K, Miyagoe Y, Ishikawa A, Hanaoka K (2002). Quantitative evaluation of expression of iron-metabolism genes in ceruloplasmin-deficient mice.. Biochim Biophys Acta.

[39] Yoshida K, Kaneko K, Miyajima H, Tokuda T, Nakamura A (2000). Increased lipid peroxidation in the brains of aceruloplasminemia patients.. J Neurol Sci.

[40] Kaneko K, Nakamura A, Yoshida K, Kametani F, Higuchi K (2002). Glial fibrillary acidic protein is greatly modified by oxidative stress in aceruloplasminemia brain.. Free Radic Res.

[41] Selim MH, Ratan RR (2004). The role of iron neurotoxicity in ischemic stroke.. Ageing Res Rev (England).

[42] Millan M, Sobrino T, Castellanos M, Nombela F, Arenillas JF (2007). Increased body iron stores are associated with poor outcome after thrombolytic treatment in acute stroke.. Stroke.

[43] Soloniuk DS, Perkins E, Wilson JR (1992). Use of allopurinol and deferoxamine in cellular protection during ischemia.. Surg Neurol.

[44] Freret T, Valable S, Chazalviel L, Saulnier R, Mackenzie ET (2006). Delayed administration of deferoxamine reduces brain damage and promotes functional recovery after transient focal cerebral ischemia in the rat.. Eur J Neurosci.

[45] Oubidar M, Boquillon M, Marie C, Schreiber L, Bralet J (1994). Ischemia-induced brain iron delocalization: Effect of iron chelators.. Free Radic Biol Med.

[46] Prass K, Ruscher K, Karsch M, Isaev N, Megow D (2002). Desferrioxamine induces delayed tolerance against cerebral ischemia in vivo and in vitro.. J Cereb Blood Flow Metab.

[47] Weinreb O, Amit T, Mandel S, Kupershmidt L, Youdim MB (2010). Neuroprotective multifunctional iron chelators: from redox-sensitive process to novel therapeutic opportunities.. Antioxid Redox Signal.

[48] Castellanos M, Puig N, Carbonell T, Castillo J, Martinez J (2002). Iron intake increases infarct volume after permanent middle cerebral artery occlusion in rats.. Brain Res.

[49] MacMillan V, Fridovich I, Davis J (1993). Failure of iron chelators to protect against cerebral infarction in hypoxia-ischemia.. Can J Neurol Sci.

[50] Schäbitz WR, Sommer C, Zoder W, Kiessling M, Schwaninger M (2000). Intravenous brain-derived neurotrophic factor reduces infarct size and counterregulates Bax and Bcl-2 expression after temporary focal cerebral ischemia.. Stroke.

[51] Zhang Y, Pardridge WM (2001). Neuroprotection in transient focal brain ischemia after delayed intravenous administration of brain-derived neurotrophic factor conjugated to a blood-brain barrier drug targeting system.. Stroke.

[52] Shi Q, Zhang P, Zhang J, Chen X, Lu H (2009). Adenovirus-mediated brain-derived neurotrophic factor expression regulated by hypoxia response element protects brain from injury of transient middle cerebral artery occlusion in mice.. Neurosci Lett.

[53] Cheng B, Mattson MP (1994). NT-3 and BDNF protect CNS neurons against metabolic/excitotoxic insults.. Brain Res.

[54] Mattson MP, Lovell MA, Furukawa K, Markesbery WR (1995). Neurotrophic factors attenuate glutamate-induced accumulation of peroxides, elevation of intracellular Ca2+ concentration, and neurotoxicity and increase antioxidant enzyme activities in hippocampal neurons.. J Neurochem.

[55] Gratacòs E, Pérez-Navarro E, Tolosa E, Arenas E, Alberch J (2001). Neuroprotection of striatal neurons against kainate excitotoxicity by neurotrophins and GDNF family members.. J Neurochem.

[56] Mattson MP (2010). The impact of dietary energy intake on cognitive aging.. Front Aging Neurosci.

[57] Schabitz WR, Schwab S, Spranger M, Hacke W (1997). Intraventricular brain-derived neurotrophic factor reduces infarct size after focal cerebral ischemia in rats.. J Cereb Blood Flow Metab.

[58] Ferrer I, Krupinski J, Goutan E, Marti E, Ambrosio S (2001). Brain-derived neurotrophic factor reduces cortical cell death by ischemia after middle cerebral artery occlusion in the rat.. Acta Neuropathol.

[59] Zhang Y, Pardridge WM (2006). Blood-brain barrier targeting of BDNF improves motor function in rats with middle cerebral artery occlusion.. Brain Res.

[60] Zhao LR, Risedal A, Wojcik A, Hejzlar J, Johansson BB (2001). Enriched environment influences brain-derived neurotrophic factor levels in rat forebrain after focal stroke.. Neurosci Lett.

[61] Endres M, Gertz K, Lindauer U, Katchanov J, Schultze J (2003). Mechanisms of stroke protection by physical activity.. Ann Neurol.

[62] Arumugam TV, Phillips TM, Cheng A, Morrell CH, Mattson MP (2010). Age and energy intake interact to modify cell stress pathways and stroke outcome.. Ann Neurol.

[63] Ke Z, Yip SP, Li L, Zheng XX, Tong KY (2011). The effects of voluntary, involuntary, and forced exercises on brain-derived neurotrophic factor and motor function recovery: a rat brain ischemia model.. PLoS One.

[64] Chen J, Zacharek A, Zhang C, Jiang H, Li Y (2005). Endothelial nitric oxide synthase regulates brain-derived neurotrophic factor expression and neurogenesis after stroke in mice.. J Neurosci.

[65] Ploughman M, Windle V, MacLellan CL, White N, Dore JJ (2009). Brain-derived neurotrophic factor contributes to recovery of skilled reaching after focal ischemia in rats.. Stroke.

[66] Rutherford LC, Nelson SB, Turrigiano GG (1998). BDNF has opposite effects on the quantal amplitude of pyramidal neuron and interneuron excitatory synapses.. Neuron.

[67] Sakata K, Woo NH, Martinowich K, Greene JS, Schloesser RJ (2009). Critical role of promoter IV-driven BDNF transcription in GABAergic transmission and synaptic plasticity in the prefrontal cortex.. Proc Natl Acad Sci U S A.

[68] Puig S, Askeland E, Thiele DJ (2005). Coordinated remodeling of cellular metabolism during iron deficiency through targeted mRNA degradation.. Cell.

[69] Wang J, Pantopoulos K (2011). Regulation of cellular iron metabolism.. Biochem J.

[70] Pinero DJ, Li NQ, Connor JR, Beard JL (2000). Variations in dietary iron alter brain iron metabolism in developing rats.. J Nutr.

[71] Kruman I, Bruce-Keller AJ, Bredesen D, Waeg G, Mattson MP (1997). Evidence that 4-hydroxynonenal mediates oxidative stress-induced neuronal apoptosis.. J Neurosci.

[72] Zhang J, Yang ZJ, Klaus JA, Koehler RC, Huang J (2008). Delayed tolerance with repetitive transient focal ischemic preconditioning in the mouse.. Stroke.

